# Introduction of male circumcision for HIV prevention in Uganda: analysis of the policy process

**DOI:** 10.1186/s12961-015-0020-0

**Published:** 2015-06-20

**Authors:** Walter Denis Odoch, Kenneth Kabali, Racheal Ankunda, Joseph Mumba Zulu, Moses Tetui

**Affiliations:** East, Central and Southern Africa-Health Community (ECSA), P.O. Box 1009, Arusha, Tanzania; African Centre for Health System Strengthening Innovations (Afri-CHEST), P.O. Box 367251, Kampala, Uganda; Uganda Protestant Medical Bureau, Plot 877, Balintuma Road, P.O. Box 4127, Kampala, Uganda; Ernest Cook Ultrasound Research Education Institute, Sir Albert Cook Building, Mengo Hospital, P.O. Box 7161, Kampala, Uganda; Department of Public Health, School of Medicine, University of Zambia, P.O. Box 50110, Lusaka, Zambia; Umeå International School of Public Health (UISPH), Umeå University, SE 90185 Umeå, Sweden; Makerere University School of Public Health, New Mulago Hospital Complex, P.O. Box 7072, Kampala, Uganda

**Keywords:** Context, HIV prevention, Male circumcision, Policy analysis, Policy process, Uganda

## Abstract

**Background:**

Health policy analysis is important for all health policies especially in fields with ever changing evidence-based interventions such as HIV prevention. However, there are few published reports of health policy analysis in sub-Saharan Africa in this field. This study explored the policy process of the introduction of male circumcision (MC) for HIV prevention in Uganda in order to inform the development processes of similar health policies.

**Methodology:**

Desk review of relevant documents was conducted between March and May 2012. Thematic analysis was used to analyse the data. Conceptual frameworks that demonstrate the interrelationship within the policy development processes and influence of actors in the policy development processes guided the analysis.

**Results:**

Following the introduction of MC on the national policy agenda in 2007, negotiation and policy formulation preceded its communication and implementation. Policy proponents included academic researchers in the early 2000s and development partners around 2007. Favourable contextual factors that supported the development of the policy included the rising HIV prevalence, adoption of MC for HIV prevention in other sub-Saharan African countries, and expertise on MC. Additionally, the networking capability of proponents facilitated the change in position of non-supportive or neutral actors. Non-supportive and neutral actors in the initial stages of the policy development process included the Ministry of Health, traditional and Muslim leaders, and the Republican President. Using political authority, legitimacy, and charisma, actors who opposed the policy tried to block the policy development process. Researchers’ initial disregard of the Ministry of Health in the research process of MC and the missing civil society advocacy arm contributed to delays in the policy development process.

**Conclusions:**

This study underscores the importance of securing top political leadership as well as key implementing partners’ support in policy development processes. Equally important is the appreciation of the various forms of actors’ power and how such power shapes the policy agenda, development process, and content.

## Background

Health policy analysis, especially the analysis of actors’ power and process is important in understanding why a policy succeeds or fails [1]. Analysis of power in the policymaking process is essential because of their complex and political nature. The process is often complex because the actors’ power or position in the political hierarchy may play a more important role in shaping both the process and content of health policy reform than their knowledge and understanding of the issue [2].

The analysis of power and process provides insight into policy actors’ interests in, positions on, and power to set the policy agenda and shape the policy process and content [3]. Information from such analyses is critical in developing viable health policy proposals, but such analyses have rarely been conducted in low- and middle-income countries (LMICs) [4, 5]. Further, the limited literature on health policy analyses in LMICs concentrates much on policy content despite the central role that power and process play in determining policy change and reforms [6, 7].

This study therefore aimed at generating additional knowledge about health policy processes and power interactions amongst various stakeholders, particularly for policies focusing on HIV prevention. We focused on male circumcision (MC), a relatively new HIV prevention innovation, to examine how actors’ power influence the policymaking process in Uganda.

In 2007, three randomised controlled trials (RCTs) showed that MC can reduce the heterosexual acquisition of HIV infection in men by approximately 60 % [8–10]. Following this evidence, the World Health Organization (WHO) recommended MC to be considered as part of comprehensive HIV prevention strategies in countries with high HIV and low MC prevalence [11]. Fourteen countries in sub-Saharan Africa, including Uganda, were identified as priority countries that could benefit from a MC program for HIV prevention [12].

In 2006, Uganda’s Ministry of Health (MoH) estimated MC prevalence at 25 %, which was among the lowest in sub-Saharan Africa; however, the country’s HIV prevalence at 6.5 % was among the highest in sub-Saharan Africa [13]. In this analysis, we chose Uganda particularly because of the very important and useful lessons drawn globally from its previous HIV prevention policy success among LMICs [14]. We hope that the country’s MC policy development experience adds value to those attempting to develop similar policies or understand similar policy processes. Herein, we identified actors and examined their power and how they used it to influence the MC policy process.

## Methods

### Conceptual framework

The ‘policymaking process’ refers to the way in which policies are initiated, developed or formulated, negotiated, communicated, implemented, and evaluated [15]. This process often occurs in stages, which include problem identification and issue recognition, policy formulation, implementation, and policy monitoring and evaluation [15]. Two frameworks are used in this paper to analyse the policy process of the introduction of MC for HIV prevention in Uganda. First, a framework demonstrating the relationship of the key concepts in the policy development and, second, a framework for analysis of actors’ influence on MC policy process.

#### Framework demonstrating the relationship of the key concepts in policy development

**Fig. 1:**
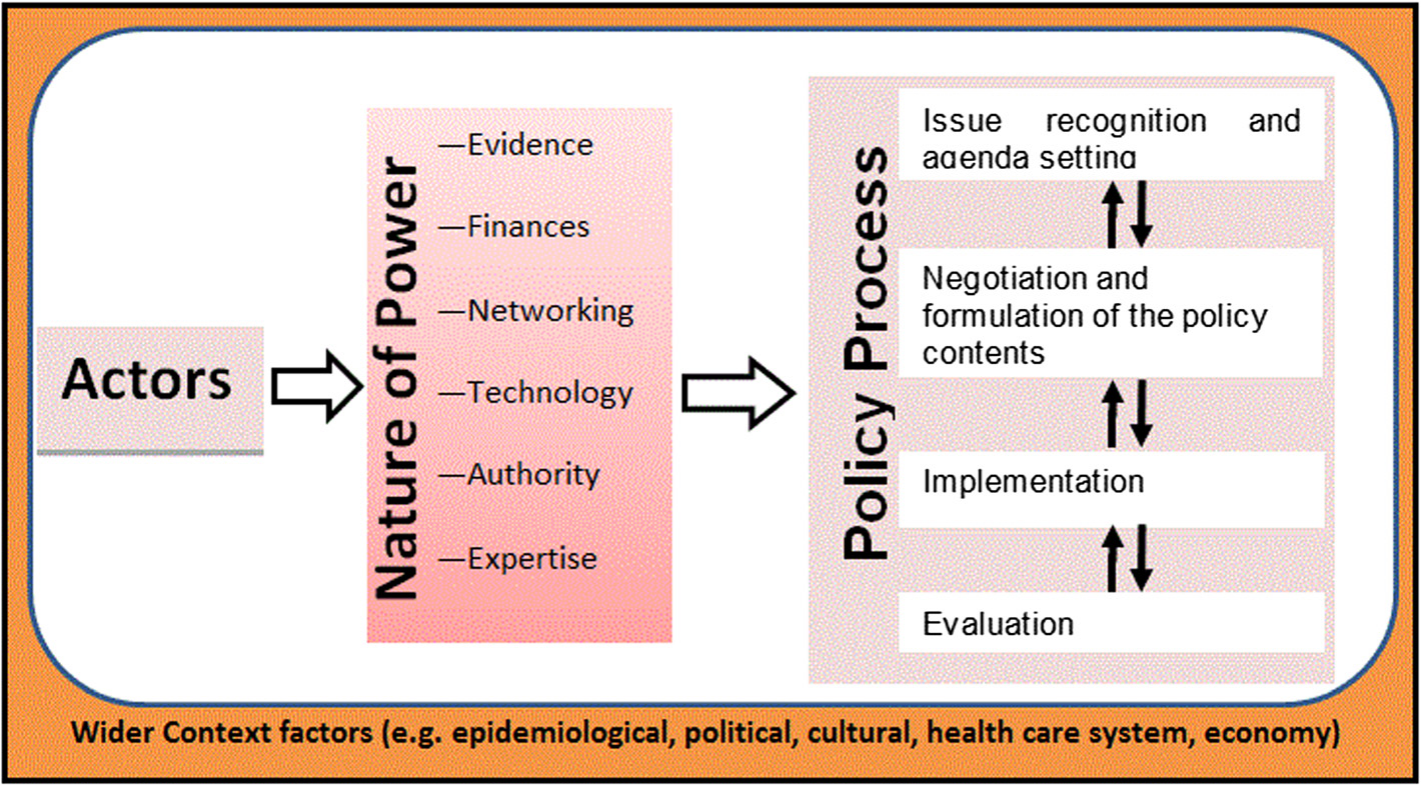
Conceptual framework for understanding male circumcision policy process in Uganda

A framework demonstrating the interrelationship of the key elements in the MC policy process is shown in Fig. 1. The framework was conceived from Walt and Gilson’s concepts for analysing the interrelationships between actors, process, and contexts [6]. The framework shows the linkage between actors and the form of power used by actors to influence the policymaking process. The framework also shows the interplay that takes place within a medium (context) that commonly affects the power wielded by actors. This context often influences the outcome of the process. Lindblom and Woodhouse, cited in Buse et al. [16], noted that the policymaking process is not linear and the form of power and extent by which an actor wields power varies with time and the stage at which the process is at. However, stages heuristics (agenda setting, negotiation and content formulation, communication and implementation, and evaluation) is important in simplifying of the policymaking process stages, despite the possibility of the process moving back and forth [15].

In this study, Kingdon’s multiple stream theory is also used to highlight how MC became an international and national agenda [17]. The theory is based on the precept that there must be a convergence of three independent streams of problems, policy, and politics for an issue to reach a policy agenda. We thus identified these streams and highlighted how they converged and ensured that MC reached the national policy agenda.

#### Framework for analysis of actors’ influence

Foucault’s [18] concept of power and the concept of position mapping of actors such as that used by Glassman et al. [3] informed the actors’ analysis framework, where actors are identified, and their position, nature and magnitude of power and level of commitment established. An actor in the MC policymaking process in Uganda was an individual or organization with interest in the MC policy, and participated in the process either overtly or covertly. Actors who seek to influence the policymaking process usually deploy their powers in accordance with their position, interest, and commitment to the policy [16].

**Table 1:** Keyword and number of hits obtained

Search number	Keyword	Number of hits
1	Male.mp	92,879
2	HIV.mp	111,728
3	AIDS.mp	57,454
4	Circumci*.mp	952
5	Prevent*.mp	171,079
6	Polic*.mp	45,346
7	Uganda.mp	4,952

* implies truncation of keywords, mp implies searching for keywords in abstract, title, original title, broad terms, heading words

**Table 2:** Logical combinations of the keywords in Table 1 were made to narrow the number of articles

Search number	Keyword combination	Number of hits
8	Male and Circumci* (1&4)	482
9	HIV or AIDS (2 or 3)	124,467
10	Prevent* and (Male and Circumci*) and (HIV or AIDS)	236
11	Polic* and search number 10	42
12	Uganda and search number 11	5

**Table 3:** Some of the stakeholders meetings with male circumcision on the agenda

Conference/Meeting	Organizer/Funder	Date (location)
	National Level	
Meeting the Demand for Male Circumcision	Forum for Collaborative HIV Research in collaboration with the bill and Melinda gates foundation, WHO, and UNAIDS	13–14/Mar/2008, (Kampala, Uganda)
National stakeholders’ meeting on safe male circumcision organized by Family Health International (FHI)	FHI and African Medical and Research Foundation	8/Dec/2007, (Kampala, Uganda)
HIV/AIDS Implementers’ Meeting	Hosted by Government of Uganda and sponsored by United States President’s Emergency Plan for AIDS Relief, UNAIDS, the World Bank and WHO	3–7/Jun/ 2008 (Kampala, Uganda)
	International Level	
Consultation Modelling the Impact of Male Circumcision on HIV Transmission	UNAIDS and WHO	17–18/ Nov/2005, (Geneva)
Male circumcision: current epidemiological and field evidence; program and policy implications for HIV prevention and reproductive health	United States Agency for International Development	September 18, 2002, (Washington, DC)
Conference on Retroviruses and Opportunistic Infections (CROI)	CROI	Feb/2006 (Denver, United States)
Regional Consultation on Safe Male Circumcision and HIV Prevention	UN Regional Working Group on Male Circumcision	20–21/Nov/2006 (Nairobi, Kenya)
Meeting on strategies and approaches for male circumcisionprogramming	WHO	5–6 /Dec/ 2006, (Geneva)
Male Circumcision and HIV Prevention: Operations Research Implications. An International Consultation	WHO	21–22 June 2007, (Nairobi, Kenya)

Sources of data [25, 32, 56]

Power is generally considered as the ability to influence other actors to agree with your position [16]. However, within the social arena where policymaking processes occur, power is a fluid concept, consisting of force relationships operating in a system with effects on social institutions and controls. According to Foucault [18], power must be understood first as the multiplicity of force relations intrinsic in the sphere in which they operate and which constitute their own organization. Secondly, as the process that transforms, strengthens, or even reverses a policymaking process through ceaseless struggles and confrontations. Thirdly, as the support that policymaking actors find in each other, thus forming a chain or a system, or on the contrary, the disjunctions and contradictions which isolate them from one another. Finally, as the strategies which policymaking actors use, whose general design or institutional crystallization is embodied in the state apparatus, in the formulation of the law, in the various social hegemonies.

As Foucault argues [18], power is many forces relating to each other within a system and in this study, these forces are considered as the forms of power. The forms of power in the MC policymaking process are knowledge (expertise), financial stature, legitimacy, authority, networking capability, technology, and structural organization [16, 19, 20]. Although there are relationships in these forms of power, their distribution amongst actors is usually not uniform and thus the usual polarization between proponents and opponents on a policy issue.

### Data collection and analysis

Secondary data was used in the analysis of the MC for HIV prevention policy process in Uganda. No ethical clearance was sought because the information used in this study was publically available and the study did not involve human subjects.

Sources of data included hard copy documents on MC for HIV prevention and other relevant documents such as HIV policies, programmes and strategic plans, relevant meeting minutes, and published articles from Uganda Government institutions notably Uganda AIDS Commission and MoH, and United Nations institutions mainly WHO and UNAIDS. Soft copies of documents relevant to the research topic were obtained through searching of the websites of the MoH, WHO, and UNAIDS and the electronic archives of the two main newspaper companies in Uganda – the New Vision and the Daily Monitor. Additionally, electronic databases PubMed, Global Health, Web of Science, and Popline were also searched. The key words used during the search processes are indicated under each database searched below. Truncation was used during the search in order to identify keywords, even if the word was used in different tenses.

Keywords searched, combinations, and number of hits from Global Health database are as indicated in Tables 1 and 2.

A similar search strategy described under Global Health database was used in searching Popline, PubMed, and Web of Science databases. Popline database yielded 47 relevant articles using the final combination of Male & circumcision* & HIV/AIDS & prevention* & Uganda & policy*. PubMed yielded seven articles in the final advance searched using ((Male AND Circumcision) AND (HIV OR AIDS) AND (Prevention) AND (Uganda) AND policy). Finally, Web of Science database yielded 14 articles using the final combination of Male circumcision* AND HIV prevent* AND Uganda AND Polic*.

There were overlaps in the hits (articles) yielded, i.e., at least each hit appeared in two or more of the searched databases. Thus, the search of the four databases was deemed sufficient, as no new relevant articles were obtained from searching additional databases.

Restricting the search to peer reviewed and journal published articles was deemed inappropriate as this would prevent other reports, such as conference and workshop reports, from appearing during the search; conference, workshop, and meeting reports are important in understanding particular policy development processes. Given the non-sophistry of the newspaper electronic archives, the word search was only circumcision and it yielded 16 articles from both newspaper archives.

During the reviews of the identified relevant articles from the above searches, other articles relevant to the study topic were identified through a snowballing technique. That is, relevantly cited articles in the article being reviewed were searched using search engines, Google Scholar, and Scirus.

The inclusion and exclusion criteria used to further narrow down the number of articles to only those relevant to the answering of the study objective were a) inclusion: availability of full length article, publication in English, and publication between 1990 and 2012 (background reading had indicated that the issue of MC in relation to HIV prevention started in the mid-1990s), and b) exclusion: non-English articles, non-accessibility of full article, and non-relevancy to the research objective, such as those discussing mainly circumcision bioscience. In total, we reviewed 230 relevant documents and used 153 documents in the final analysis.

Relevant information from the articles was summarised and organized in themes. In the analysis, themes were framed with regards to the study objectives. This is “a method for identifying, analysing and reporting patterns (themes) within data. It organizes and describes data set in detail and goes further to interpret various aspects of the research topic” ([21], p. 79).

The themes included actors, form of power, and context. In the actor theme, the analysis included the identification of actors at each of the MC policy process stages. Whereas in the form of power theme, the magnitude of an actor’s power in influencing a MC policy process stage was graded from a minimum (+) to a maximum (+++). The magnitude of power was deduced based on the extent of the actor’s influence in the policy process using available information. Direct quotations from the data sources were used in shedding more light on these inferences. The position of actors were also inferred from the available information as supportive, non-supportive, conditionally supportive, opposing, mixed, and neutral, while the level of commitment was either none, low, moderate, or high.

Under the context theme, information relating to the general contextual factors that affect HIV/AIDS response and new policies were summarised. One of the study limitations was inability to access minutes of National Task Force on MC meetings. The National Task Force minutes would have added value in the making of inferences, for example, on the position and power of some actors. However, by accessing the newspaper articles, and other published reports, the effects of unavailability of these minutes on drawing of deductions were minimised. Interviewing actors or their representatives and triangulating it with the secondary data would also have added value in making deductions. However, we believe that the robustness with which the secondary data were collected and analysed, including involvement of more than one person in these processes, makes this study a worthwhile contribution to the field of health policy analysis.

## Results and discussion

### Main events in the MC policy process in Uganda

Review of the literature showed that a linear construction of the MC policy process in Uganda included putting the MC for HIV prevention on the national policy agenda, negotiation and formulation, and communication and implementation. No information could be found on the MC policy evaluation. The lack of information on evaluation is perhaps because the policy on MC only came to effect in 2010.

To appreciate how MC for HIV prevention became a national issue in Uganda, it is prudent to first understand how it gained international recognition. The link between HIV infection and MC emerged out of observations that certain regions in Africa had higher HIV prevalence compared to others [22]. In a systemic review and meta-analysis by Weiss et al. [22], 21 of the 27 observational studies included in the analysis showed up to 50 % reduced risk of HIV infection amongst circumcised men. Internationally, the advocates of MC for HIV prevention were largely researchers. Evidence from these scientific studies was essential in shaping the policy process.

The frequently appearing researchers on MC in relation to HIV prevention included Kawango Agot, Bertran Auvert, Robert C Bailey, John Bongaarts, Ronald H. Gray, Daniel Halperin, Godfrey Kigozi, Stephen Moses, David Serwadda, Maria J. Wawer, and Thomas C. Quin. In fact, 80 % of the 42 hits from Global Health database search were authored by at least one of these researchers. By presenting their findings based on observational studies at international conferences, they urged WHO and UNAIDS to recommend MC to be included as part of a global HIV/AIDS response. However, it is palpable that advocacy based on observational studies by the researchers on the inverse relation of MC and HIV prevalence was insufficient to persuade WHO and UNAIDS to recommend it to countries. This is attested to by the fact that UNAIDS and WHO waited until results from three RCTs were out before issuing policy recommendations in 2007 [12].

A number of sub-Saharan countries developed their MC policies immediately following UNAIDS and WHO recommendations [23]. There was also a relatively short gap between publication of the first RCT result and the development of the UN work plan on MC; the UN work plan on MC was available by 2005 [24]. It is therefore conceivable that this first RCT study coupled with earlier observational studies (policy stream), the lobbying and activism by academic researchers and international non-government organizations (NGOs) involved in the HIV/AIDS response (politics stream), and the sustained high HIV prevalence in sub-Saharan Africa despite the Abstinence, Be faithful and use Condoms strategy (problem stream) led to MC gaining international recognition in the second half of the last decade.

**Fig. 2:**
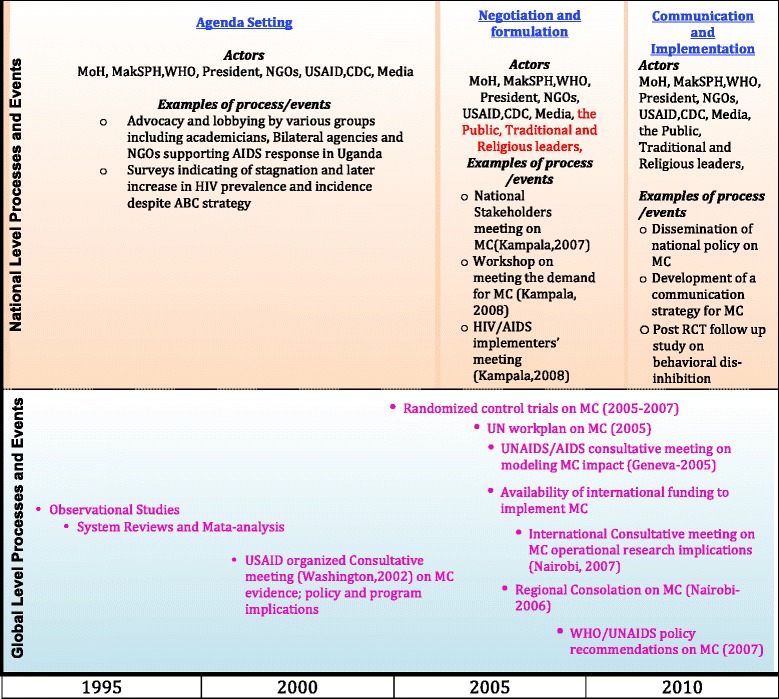
Illustration of some of the events and processes in the development of the male circumcision policy

Convergence of Kingdon’s politics, policy, and problem streams [17] was also observable at the national level. With MC for HIV prevention firmly on the international policy agenda and lobbying by national and international NGOs (politics stream), the rising HIV prevalence in Uganda (problem stream), and evidence that MC was a significant additional ‘armament’ to the HIV fight (policy stream), MC soon became a national issue in Uganda. Between 2005 and 2008, a number of stakeholder meetings were held where MC was the main agenda. Table 3 shows examples of the stakeholder meetings where MC for HIV prevention was discussed and Fig. 2 also depicts some of the major events and processes at national and international level.

**Table 4:** Major actors during male circumcision policy agenda setting

Actor analysis category	Organization name
MoH	Ministry of Health-Uganda
The President	The President of Uganda
US agencies	United States Agency for International Development, PEPFAR, CDC
UN agencies	WHO and UNAIDS
NGOs	Baylor College Uganda Management Science for Health, Elizabeth Glazer’s Paediatric AIDS Foundation, Infectious Diseases Institute-Kampala, United States Walter Reed, Family Health International
MakSPH	The academic researchers from Makerere University School of Public Health
Media	The New Vision and the Daily Monitor

MakSPH, Makerere University School of Public Health; MoH, Ministry of Health; NGO, Non-governmental organizations; PEPFAR, President’s Emergency Plan for AIDS Relief; UN, United Nations; US, United States of America; CDC, US Centres for Disease Control and Prevention. Source of data: [52]

Despite initial paucity by the MoH in Uganda, by early 2008, it took up efforts in coordinating the MC policy process [25]. The MoH had the support of a number of partners involved in the HIV/AIDS response in Uganda, who wanted the MC implemented as early as 2007. Nonetheless, other actors, such as Christian religious leaders, did not even want it considered [26]. Table 4 shows the main actors at the agenda setting stage of the MC policy process.

MC for HIV prevention in Uganda gained greatest momentum from the HIV/AIDS development partners’ efforts and researchers. The MoH, for example, claims it was not involved or informed of the initiation of the MC clinical trial in Uganda. An official from the MoH was quoted as saying:

“*We didn’t even know that this study was going on. Several research institutions partner with funders and conduct research… This is one of the researches that* [were] *conducted without much of our knowledge*” [27].

Most partners supporting HIV/AIDS response argued that a policy was essential to guide support for MC activities and funding, the MoH needed to be more involved [28]. However, the MoH still felt that, even though the MC evidence was compelling, more information was needed. A MoH official reflected this in a public debate speech in March 2008, when he said, “*the government will only come up with a national policy on male circumcision as one of the measures to minimise HIV infection after consulting widely*” [29]. The President of Uganda also did not support MC becoming an HIV prevention method, let alone having a specific policy on MC [30, 31]. The President was concerned that MC roll-out would lead to behavioural dis-inhibition and undo the gains made in the fight against HIV/AIDS.

The MoH organized a preliminary HIV/AIDS partners’ meeting in 2007. During this initial meeting, development partners committed to supporting studies that would help answer some of the issues being raised about MC such as its effects on sexual behaviours, acceptability amongst the populace, mechanism of integrating MC into other health programs, and the resource implications. An acceptability and feasibility study commissioned by the MoH in 2007 found, among others, that political, religious, cultural, and opinion leaders have most influence on people and could publicly support MC if they clearly understood MC and were sure it did not promote immorality [32]. Apart from the initial concern by the MoH regarding the manner in which the MC studies were conducted i.e., without MoH involvement, it was also concerned with the development of a specific policy on MC. This is because for MC to have effect on the HIV infection rate, it requires it to be implemented on a large scale, yet there was limited capacity of health facilities to handle MC on that scale. In addition, there were also legal concerns about traditional and Muslim circumcisers continuing to circumcise once a MC policy came to effect.

This process of getting more information and dealing with opposition from a section of actors, including the President, delayed the process of developing the MC policy document. It lasted from 2007, when it became a national issue, to late 2010 when the policy document was made and communicated.

### The power interaction amongst actors and contextual factors that affected the actors’ expression of influence during the policy process

The MC policy process in Uganda generally took a longer course compared to other countries such as Lesotho, Zambia, and South Africa, who developed their policies on MC in the immediate aftermath of WHO and UNAIDS recommendations. The delay in Uganda may be attributed to what Foucault [18] describes as ‘polarization’ amongst actors on a policy issue. The proponents and opponents used their power to affect the MC policy process. The influence variables (magnitude of power, form of power, position, and level of commitment) of each of the actors during each of the MC policy process stages are summarized in Tables 5,6,7. The influence variables of the actors were deduced from information collected as described in the methods section.

**Table 5:** Framework for analysis of actor influence (putting MC for HIV prevention on the national policy agenda)

Actor	Position on male circumcision for HIV prevention	Magnitude of power	Main nature of power	Level of commitment
MoH	Non-supportive	+++	Legal authority	Low
MakSPH	Support	+	Evidence (expertise), networking ability	High
UN	Support	++	Legitimacy, structural organization, expertise, networking ability	High
The President	Oppose	+++	Political authority, legitimacy	Low
NGO	Support	+	Networking ability, financial	High
US agencies	Support	+++	Financial expertise	High
Media	Neutral	++	Public communication	Low

MakSPH, Makerere University School of Public Health; MoH, Ministry of Health; NGO, Non-governmental organizations; UN, United Nations; US, United States of America

**Table 6:** Framework for analysis of actors’ influence (negotiation and formulation of MC policy)

Actor	Position on male circumcision for HIV prevention	Magnitude of power	Main nature of power	Level of commitment
MoH	Conditionally supportive	+++	Authority, legitimacy	Medium
MakSPH	Supportive	+	Expertise, evidence	High
UN	Supportive	++	Legitimacy, structural organization, expertise, networking ability	High
The President	Oppose	+++	Authority (political) legitimacy	Low
NGOs	Supportive	+	Networking financial	High
US agencies	Supportive	+++	Financial expertise	High
Media	Neutral	++	Public communication	Medium
Traditional leaders (circumcising areas)	Conditionally supportive	+	Traditional authority	Low
Traditional leaders (non-circumcising areas)	Oppose	+	Traditional authority	Low
Religious leaders (non-Muslims)	Oppose	+	Charismatic authority	Low
Muslim leaders	Conditionally supportive	+	Charismatic authority	Low
Public	Mix	++	Numbers	Low

MakSPH, Makerere University School of Public Health; MoH, Ministry of Health; NGO, Non-governmental organizations; UN, United Nations; US, United States of America

**Table 7:** Framework for analysis of actors’ influence (communication and implementation)

Actor	Position on male circumcision for HIV prevention	Magnitude of power	Main nature of power	Level of commitment
MoH	Supportive	+++	Authority, legitimacy	High
MakSPH	Supportive	+	expertise, evidence	High
UN	Supportive	++	Legitimacy, structural organization, expertise, networking ability	High
The President	Non-supportive	+++	Authority (political) legitimacy	Low
NGOs	Supportive	+++	Networking financial	High
US agencies	Supportive	+++	Financial expertise	High
Media	Neutral	++	Public communication	High
Traditional leaders (circumcising areas)	Supportive	+	Traditional authority	Low
Traditional leaders (non-circumcising areas)	Oppose	+	Traditional authority	Low
Religious leaders (non-Muslims)	Oppose	+	Charismatic authority	Low
Muslim leaders	Supportive	+	Charismatic authority	Low
Public	Mix	++	Numbers	Low

MakSPH, Makerere University School of Public Health; MoH, Ministry of Health; NGO, Non-governmental organizations; UN, United Nations; US, United States of America

#### Issue recognition and agenda setting for MC for HIV prevention in Uganda

Table 5 shows the main actors and power variables of each actor during the policy process stage of issue recognition and agenda setting, while Table 4 shows how some of these actors were grouped. For example, United States (US) agencies in this paper refers to any of USAID, CDC, and PEPFAR. At this stage, the MoH position was described as non-supportive and with a low level of commitment as reflected in a statement by an official from the MoH:

“*Here was someone claiming to have conducted a study in Rakai on male circumcision and found it to have protective chances. We want more details given that we didn’t discuss the proposal, didn’t know the methodology…*” [27].

In large institutions such as the MoH, individuals may have differences of opinion; however, what is publicly stated should be considered the organization’s position [16]. The MoH as a lead agency in the health sector is powerful and this is expressed in its legal authority recognized in the constitution of Uganda [33]. In addition, the government, through the MoH, is the main provider of health services in the country and implementation of MC has inevitable metastatic effects on other health care system aspects. For such a policy with probable system-wide effects, the MoH’s support and high level of commitment is needed for it to succeed at any of the policy process stages. Therefore, both the proponents and the opponents courted the MoH as a key actor and the direction of its eventual leaning was important for the MC policy to reach the national policy agenda. The courting of the MoH can be seen in the President’s speeches regarding MC, where he continually discouraged the MoH from wasting efforts on the MC issue [31]. Proponents, including NGOs and US agencies, as noted earlier, facilitated the MoH in commissioning researches as well as funding MC workshops.

As the authors of the RCT in Uganda, the Makerere University School of Public Health (MakSPH) was supportive of the policy at all the stages of the policy process. However, the magnitude of MakSPH power in influencing how the MC issue reached a national agenda seemed limited. The MakSPH in its own admittance of its low level of influence on national MC policy issue, stated in one of its publication that, “*the evidence around male circumcision prioritized global agencies relative to national ones, and national level stakeholders were treated as secondary audiences*” [34]. This might be one reason for the slow uptake of evidence and the delayed policy process in Uganda. The United Nations (UN) derives its power by its structural organisation, networking capability, expertise represented by WHO and UNAIDS, and is also perceived to work for public good, thus the legitimate power.

The President’s power is the political authority as enshrined in the National constitution [33]. In the fight against HIV/AIDS, the President is also globally recognized [35]. Therefore, in policy issues around HIV/AIDS, the President has strong legitimate influence and thus a high power. As noted by Cocks cited in Boyle and Hill [p. 328, 33], the President was opposed to stressing MC as a major HIV prevention strategy. Although the President remained opposed to the MC policy process, he did to commit specific resources in opposing MC. This may be related to the change in position of the MoH, as described in the following policy process stages, but it could also be attributed to the existence of scientific evidence from the RCTs being embraced by other countries. Therefore, despite his high level of power, the President’s level of commitment may be described as low during the MC policy process stages.

The US agencies supported MC policy for HIV prevention. This is reflected in funding of studies such as the feasibility and acceptability of MC and the modelling of economic impact of MC in Uganda. The power of US agencies is reflected in their expertise and financial status. More than 80 % of the external funds for the HIV/AIDS response in Uganda are from the US agencies and external funds constitute over 90 % of Uganda’s HIV/AIDS response budget and expenditure [36]. The NGOs were supportive of the MC policy, organizing workshops and piloting MC implementation. However, their powers in influencing MC policy seem to have relied greatly on their strong link with UN and US agencies. On the whole, the media position on MC may be described as neutral, but they were quite powerful in getting information to the public at a fast speed and shaping public opinion.

#### Negotiation and formulation of MC policy

At this policy stage, additional new actors emerged, including traditional and religious leaders and the general public. The actors’ influence variables at this stage are shown in Table 6. The position, magnitude of power, and level of commitment of the MakSPH, UN, the President, NGOs, US agencies, and the media remained the same as in the stage of issue recognition and agenda setting. The MakSPH conducted a follow-up study in 2008 and the report indicated no difference in sexual behaviour between circumcised and uncircumcised men, thus largely negating the behavioural dis-inhibition arguments [37].

A US Agency for International Development study estimated that scaling-up MC would avert 428,000 HIV infections and save up to US$2 billion between 2009 and 2025 in Uganda [38]. This kind of evidence was important in persuading or ‘softening’ some actors who were opposed to the MC as an additional tool in the prevention of HIV. The NGOs were willing to and carried out pilot projects on MC with support from US agencies’ funding between 2008 and 2010 [23]. The MoH also became supportive, especially following the outcome of the feasibility and acceptability study on MC and the MakSPH post-RCT behavioural study. The MoH then promised to continue to engage the President on the matter [39]. Nevertheless, the President, as well as other local leaders who were supposed to promote issues such as the MC, remained opposed to MC for HIV prevention [31, 40].

The position of the traditional and religious leaders in relation to MC depended on whether they were from the circumcising areas of the country or not, and Muslim or not (Table 6). Leaders from the circumcising areas and Muslims were supportive if the policy would allow them to perform the procedure, while leaders from non-circumcising areas and non-Muslims perceived MC as a way of converting their followers to practising circumcision and promoting immorality [32]. The powers of traditional and religious leaders in influencing the policy processes stems from their traditional and charismatic authorities, respectively [16].

The traditional and religious leaders’ commitment was described as low at this stage. A search of the two main national newspapers’ archives found no joint statements by any of these groups of leaders on MC, as they usually do on other issues of national concern. The reason for the low level of commitment amongst the religious leaders was perhaps due to the change in position of the MoH to support MC given its legal and authoritative status, but also possibly due to the fact that NGOs who usually support religious institutions were in support of MC.

From the above arguments, it can be noted that, contextually, cultures are very diverse in Uganda. There are regions of the country where MC is traditionally performed, such as in the Eastern part of the country amongst the Bagisu and Sabiny tribes and also amongst Muslims. Muslims constitute about 30 % of Uganda’s population [13]. These population subgroups and their leaders were more likely to be in support of the MC [32]. However, MC is not culturally acceptable in a number of tribal groupings [41], and such groups would be against the MC. De Vincenzi and Mertens [42] argue that this kind of resentment regarding MC and other related issues make a number of African governments reluctant to agree to participate in or allow the adoption of a policy of MC. It can be argued that these aspects of stigmatization and government reluctance were in favour of MC opponents.

It is discernible that the negotiation and formulation process resulted in some compromises. For example, the technically desirable term ‘medical male circumcision’ was dropped in favour of the term ‘safe’ MC. This enabled the proponents (US agencies, UN agencies, academicians, MakSPH) to gain the support of traditional leaders from the circumcising tribes and Muslims leaders who were initially against developing a policy on MC. With the policy being called ‘safe’ as opposed to ‘medical’, these leaders would continue to perform the procedure and thus their support for the MC policy would be secured. The MoH was also in support of it being called safe, because it argued that if it were left as ‘medical’, given the prevailing health system challenges, much of the population would not access it, at least to the extent of it reaching a public health impact on HIV prevalence [43]. However, it should be noted that, during the studies on MC for HIV prevention, qualified medical professionals performed MC. Therefore, in their agreement with proponents, religious and cultural circumcisers were to continue with the practice as long as they underwent ‘safe MC’ training to circumvent some of the legal challenges.

Studies indicate that the position of the general public was mixed [32, 44]. The power of the public lies in its numbers, especially when mobilized, but this requires organization. There was no information to indicate that there were any organizations within the general public to influence the MC policy process and hence the level of commitment of the public is assumed to have been low.

**Fig. 3:**
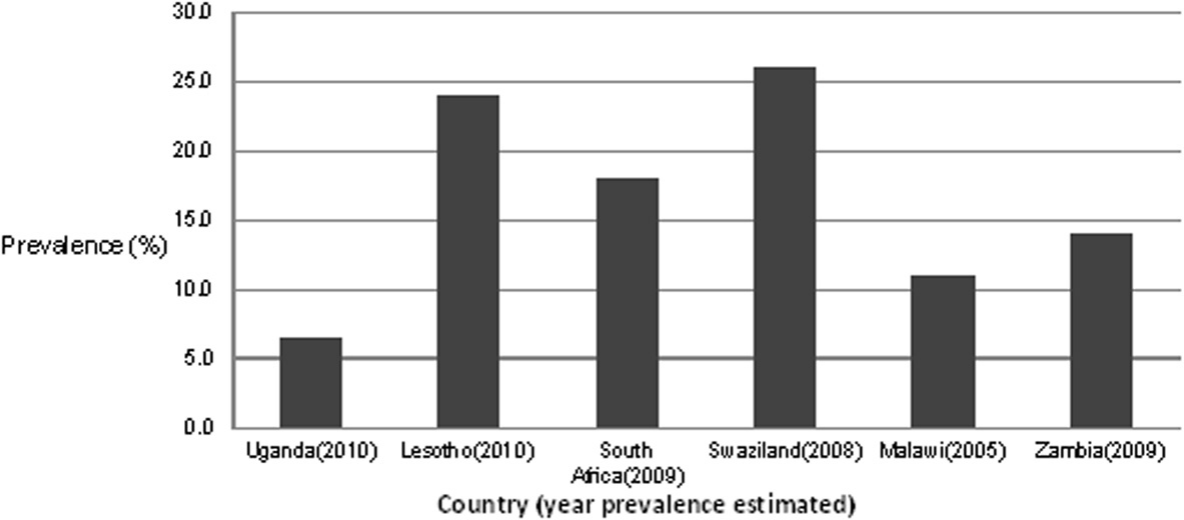
Estimated prevalence of HIV infection in selected sub-Saharan countries

The epidemiological context of HIV in Uganda also affected the speed of the MC policy process at the negotiation stage. The HIV prevalence in Uganda declined rapidly from over 25 % in the late 1980s until about 2005, when it stagnated at about 7 % [45]. The other countries that quickly considered MC still had an HIV prevalence in excess of 10 % (Fig. 3). This perhaps made it easy for proponents of MC for HIV prevention in those countries as any opponent to the development of the MC policy for HIV prevention would be viewed as being insensitive.

Non-Muslim religious leaders and the President were worried about the behavioural dis-inhibition and moral decadence with MC introduction. Recent information indicated that the main mode of transmission remains heterosexual transmission and one of the groups showing high new HIV infections were people in stable marriages [45]. People in stable marriages are traditionally assumed to exercise more fidelity. This finding coupled with stagnation in HIV prevalence and fear that it is rising despite the implementation of the old strategies, was a boost to the MC proponents and decrease in level of opposition by the President as seen in the next policy process stage.

#### Communication and implementation

The actors at this stage of the policy process remained as in the previous stage, although there were some changes in the influence variables of some actors such as that of the MoH, the President, NGOs, and religious leaders (Table 7). The MoH position at this stage was that of support and high level of commitment, stating that, “*raising the number of men who undergo the procedure* [MC] *will help the country to bring HIV prevalence down from the current 6.4 % to less than 5 % in the short term*” [43]. The President’s strong position against MC had also reduced. This is reflected in a speech during the launch of the 2010 Imbalu season [traditional circumcision season in Eastern Uganda] when he said, “*it can help* [to] *reduce it but it does not stop it*”, in reference to MC for HIV prevention [46].

The government of Uganda has historically allocated very limited resources for HIV/AIDS response activities. This makes it vulnerable to external influence and therefore willing to change its position. This is more so if the issue does not affect government existence [47]. One may argue that the MC falls under this category, since the government does not invest substantially in HIV/AIDS response. This, in addition to MoH engagement and the scientific evidence probably explain the President’s change in position from the overtly opposing to a non-supportive position.

Wakabi [43] reports that the Muslim and traditional leaders from circumcising areas of the country became more supportive because they were involved and their circumcisers were to be allowed to circumcise. However, the non-Muslim religious leaders continued to oppose MC. A newspaper reported Christian bishops saying the church cannot support MC for HIV prevention because that would be encouraging immorality and worsening HIV spread [48].

The NGOs were powerful at this stage, with many implementing the MC in most parts of the country through US agency funding. In fact over 90 % of MC performed for HIV prevention is through the NGOs, the government facilities were yet to fully embrace performing MC for HIV prevention [36]. The MakSPH remained supportive and was involved in the formulation of the MC communication strategy document [49]. The public’s position remained mixed; media reports indicated good uptake in some districts while it was shunned in others [50, 51].

The MC policy came into effect in 2010, but WHO and UNAIDS reports indicate that some MC for HIV prevention activities were already taking place, mainly implemented by NGOs with US agencies funding by 2009 [23]. Male circumcision was also mentioned as an HIV prevention strategy in a number of government documents such as the National HIV and AIDS Strategic Plan 2007/8–2011/12 [52] and the Health Sector Strategic and Investment Plan [53]. After the launch of the policy, a number of NGOs expanded their MC services to other parts of the country [54, 55]. These indicate the back and forth movement in the policy process, but with some increments in the overall process.

## Conclusions

The aim of this study was to explore how actors’ power as well as contextual factors shaped the MC policy development process in Uganda. The policymaking process was characterised by differential policy notions, power, and negotiations. The main drivers of the MC policy were largely development NGOs, funders and partners, and researchers at MakSPH. The actors who opposed the policy in the initial stages included the President and traditional and religious leaders. These actors opposed the development of the policy, as they believed that MC was not effective enough in preventing HIV, would promote immorality, and also change the cultural ways of conducting MC. The MoH, at the initial stages of the policy development process, took up the role of a neutral actor. The rising HIV prevalence and the increased scientific evidence that MC could reduce HIV transmission can explain the shift in the MoH’s position from being a neutral to an active policy development supporter. Additionally, rebranding of ‘medical’ MC to ‘safe’ MC resulted in compromise by those who opposed the development of the MC policy in the initial stages.

In general, in addition to actors’ power, the study also shows that scientific evidence, negotiations among actors, the magnitude of the problem that the policy is trying to address, as well as shaping of the policy content to reflect contextual issues play an important role in influencing the pattern of the development process and its launch.

### Study limitations

Two study limitations are highlighted under data collection and analysis sub-section. They relate to inability to conduct in-person interviews with some of the actors and the lack of review of the actual minutes of the National Task Force on Male Circumcision meetings. These would have added value to and provided more insights into the making of inferences of some of the actors’ power, commitment, position, and other variables. However, the robustness with which we collected, analyzed, and triangulated data, including involvement of all co-authors at every stage of the study, ensured that the value addition from interviews and review of other meeting minutes would not alter the study findings and conclusions. Another limitation was the inclusion of literature published only in English; non-English relevant literature might have added information, however, the effects were estimated to be low. We therefore think this study is a good addition to the field of health policy analysis, and particularly in sub-Saharan Africa where these types of studies are limited.

## Abbreviations

CDC: United States Centres for Disease Control and Prevention
LMICs: Low- and middle-income countries
MakSPH: Makerere University School of Public Health
MC: Male circumcision
MoH: Ministry of Health
NGOs: Non-government organizations
RCTs: Randomised controlled trials
UN: United Nations
US: United States
WHO: World Health Organization
